# NFL strongly correlates with TNF-R1 in the plasma of AD patients, but not with cognitive decline

**DOI:** 10.1038/s41598-021-89749-5

**Published:** 2021-05-13

**Authors:** Constance Delaby, A. Julian, G. Page, S. Ragot, Sylvain Lehmann, M. Paccalin

**Affiliations:** 1 Laboratoire de Biochimie Protéomique, INM, Université de Montpellier, INSERM, CHU Montpellier, IRMB, Montpellier, France; 2 Sant Pau Memory Unit, Department of Neurology, Institut d’Investigacions Biomèdiques Sant Pau-Hospital de Sant Pau, Universitat Autònoma de Barcelona, Barcelona, Spain; 3 EA3808-NEUVACOD Neurovascular Unit and Cognitive Disorders, University of Poitiers, Poitiers, France; 4 Memory Centers for Resources and Research, Poitiers University Hospital, Poitiers, France; 5 Centre d’Investigation Clinique CIC1402, INSERM, Poitiers University Hospital, Poitiers, France

**Keywords:** Biomarkers, Diseases, Neurology

## Abstract

Peripheral inflammation mechanisms involved in Alzheimer's disease (AD) have yet to be accurately characterized and the identification of blood biomarker profiles could help predict cognitive decline and optimize patient care. Blood biomarkers described to date have failed to provide a consensus signature, which is mainly due to the heterogeneity of the methods used or the cohort. The present work aims to describe the potential informativity of peripheral inflammation in AD, focusing in particular on the potential association between the level of plasma neurofilament light (NFL), peripheral inflammation (by quantifying IL-1β, IL-6, TNFα, CCL5, TNF-R1, sIL-6R, TIMP-1, IL-8 in blood) and cognitive decline (assessed by the MMSE and ADAScog scales) through a 2-year follow-up of 40 AD patients from the Cytocogma cohort (CHU Poitiers, Pr M. Paccalin). Our results show for the first time a strong correlation between plasma NFL and TNF-R1 at each time of follow-up (baseline, 12 and 24 months), thus opening an interesting perspective for the prognosis of AD patients.

## Introduction

Alzheimer's disease (AD) is a neurodegenerative pathology characterized by progressive cognitive impairment, leading to a loss of autonomy. In addition to the neuropathological lesions resulting from the accumulation of Aβ plaques and neurofibrillary tangles, a central and peripheral inflammatory process probably contributes to the progression of the disease^1–3^. However, the effects of inflammation may be dual, with both beneficial and detrimental sides^4^, and molecular inflammatory pathways involved in such pathological mechanisms remain to be fully characterized.

Differences in serum levels of cytokines, chemokines and growth factors have been described in patients with MCI (Mild Cognitive Impairment) or AD. However, inter-study data are often inconsistent, due in part to the heterogeneity of the population cohort used and/or the limited sensitivity of the methods employed. Thus, no blood panel has yet been identified as an aid to the diagnosis of AD and the precise role played by inflammation in neurodegeneration is still debated^1,5,6^. The possibility of identifying a panel of blood biomarkers could allow the implementation of a systematic and early diagnosis of patients at the first signs of cognitive impairment, thus optimizing their management and treatment. In addition, the prospect of the feasibility of early biochemical diagnosis of patients by a minimally invasive technique (such as venipuncture) is quite attractive. Various studies have described such a signature of biomarkers in blood: among them, Ray et al. identified 18 blood biomarkers, some of which were subsequently confirmed by ADNI^21,31^.

In particular, the role of tumor necrosis factor-alpha (TNFa) in brain neuropathology is widely described, with the pro-inflammatory cytokine being increased in plasma and cerebrospinal fluid (CSF) in AD patients^7^. Interestingly, the TNFa receptor (TNF-R1) decreases in CSF^8^ and increases in the brain^9^ and plasma^10^ of AD patients compared to control. In addition, deletion of TNF-R1 is believed to prevent cognitive deficits in AD mice through the reduction of BACE1 expression and activity^11^. On the contrary, TNFa is reported to have neuroprotective effects in the brain^12^.

Chemo attractive chemokines are known to participate in the inflammatory process of AD by modulating the migration of microglial cells to the site of inflammation^13^. In particular, interleukin-8 (IL-8, aka CXCL8) is increased following astrocyte exposure to peptides Aβ_1–42_^14,15^. We have previously shown that IL-8 concentration is higher in the CSF of AD patients than in controls^8^ and *post-mortem* studies have also reported higher IL-8 concentration in the brains of AD patients than in controls^16^. In addition, two recent meta-analyses have shown higher plasma IL-8 concentrations in AD patients than in controls^10,17^. It is noteworthy that inhibition of the IL-8 receptor in a rat model injected by Aβ_1–42_ is able to reduce local oxidative stress^18^. Soluble Aβ peptides can also trigger microglial activation in vitro and in vivo, resulting in the subsequent release of pro-inflammatory actors, such as IL-1b. This chemokine is described as elevated in CSF and brain tissue in AD patients^19^, while serum levels remain stable^8^ or elevated^20^ in AD patients compared to control, depending on the studies.

Other proteins have been described to be downregulated in the plasma of AD patients, such as CCL5 (RANTES)^21^. Matrix metalloproteinases (MMPs) and the balance between MMPs and their tissue inhibitors (TIMPs) are involved in the inflammatory regulation of AD^22^. In particular, TIMP-1 (the tissue inhibitor of MMP-9) is significantly lower in plasma of AD patients than in controls^23^, while it increases in the CSF of patients with AD and Mild Cognitive Impairment (MCI)^8,24^. Furthermore, intraventricular injection of TIMP-1 in a rodent model of AD induces a reduction in the load on Aβ and improves their cognitive function^25^. Discrepancies are described concerning IL-6 and its receptor (sIL-6R), with studies describing decrease of plasmatic IL-6^8,21^ and sIL-6R^8^ in AD patients (compared to control), and others describing a stability of this cytokine in the same context^20^.

The Neurofilament Light Chain (NFL) is a neuronal cytoskeletal protein released in the CSF and blood upon neuronal damage. A large cohort analysis revealed a positive correlation between CSF NFL and age, an association with gender (NFL being higher in males) and a negative correlation with MMSE^26–28^. It should be noted that CSF and plasma NFL are highly correlated in Down syndrome patients with AD co-pathology^29^. *Post-mortem* studies have shown a moderate correlation (r = 0.55, p < 0.001) between plasma NFL level and Braak stage in 57 AD patients, as well as a negative association between MMSE progression and plasma NFL level^30^.

In the present work, we evaluated the potential informativity of peripheral inflammation in AD: our study aims to describe the potential association between plasma NFL level, peripheral inflammation (through quantification of IL-1β, IL-6, TNFα, CCL5, TNF-R1, sIL-6R, TIMP-1, IL-8 in blood) and cognitive decline (assessed by the MMSE and ADAScog scales), through a 2-year follow-up of 40 AD patients from the Cytocogma cohort (CHU of Poitiers, Pr M. Paccalin).

## Results

### Patients’ characteristics

40 patients (10 men and 30 women) from the Cytocogma cohort were selected, based on the relative evolution of MMSE during the 2 years of follow-up: 20 patients with slow decline and 20 patients with rapid decline. Mean age of the 10 men and 30 women included in this analysis was 78 (SD = 8) years. Table 1 shows baseline neuropsychological performance and cognitive assessment scores among patient’s follow-up at 6, 12 and 24 months. Cognitive decline showed a median loss of 4 points for MMSE score. Higher scores on the ADAS-cog reflect poorer cognitive performance and results show a median increase of 6 (SD = 9) points for ADAScog score during the 24-month follow-up; 20 patients were considered as fast decliners (mean decrease of 7 points for MMSE). There was no difference between fast and slow decliners regarding age, sex, weight, Alzheimer’s disease duration or prescription of an Alzheimer’s disease symptomatic treatment at baseline and length of disease progression. Moreover MMSE and ADAScog scores did not differ significantly at baseline between the two subgroups.

**Table 1:** Clinical characteristics and neuropsychological performance at baseline and through clinical follow-up at 6, 12 and 24 months from the baseline: comparison between fast decliners and slow decliners.

	Total (n = 40)	Fast declined (n = 20)	Slow declined (n = 20)	p
Age (years), mean (SD)	78 (8)	79 (9)	77 (7)	0.534
Male gender, n(%)	10 (25%)	4 (20%)	6 (30%)	0.465
Length of disease progression (years), median [range]	2 [0–6]	2 [0–6]	3 [0–5]	0.946
Symptomatic treatment of Alzheimer’s disease at baseline, n (%)	29 (72%)	15 (75%)	14 (70%)	0.723
MMS at baseline, mean (SD)	21 (3)	21 (2)	21 (3)	0.953
ADAScog at baseline, mean (SD)	15 (6)	17 (6)	13 (5)	0.058
MMS at 6-month follow-up, mean (SD)	20 (4)	20 (4)	21 (4)	0.161
ADAScog at 6-month follow-up, mean (SD)	16 (8)	20 (8)	12 (4)	0.0004
MMS at 12-month follow-up, mean (SD)	20 (4)	18 (4)	22 (4)	0.003
ADAScog at 12-month follow-up, mean (SD)	18 (9)	23 (9)	13 (5)	0.0003
MMS at 24-month follow-up, mean (SD)	17 (5)	14 (4)	21 (3)	< 0.0001
ADAScog at 24-month follow-up, mean (SD)	21 (12)	28 (13)	15 (6)	0.0003
MMS decline (points)*, median [range]	− 4 [− 14; 3]	− 6 [− 14; − 4]	− 0.5 [− 4; 3]	< 0.0001
ADAScog decline (points)*, median [range]	2.5 [− 5; 30]	11 [− 5; 30]	− 0.1 [− 4; 11]	0.0003
MMS relative decline (%)^§^, median [range]	− 19 [− 74; 16]	− 32 [− 74; − 18]	− 2 [− 23; 16]	< 0.0001
ADAScog relative decline (%)^§^, median [range]	26 [− 29; 180]	63 [− 17; 180]	− 0.8 [− 29; 116]	0.007

*SD* standard deviation, *MMSE* Mini-Mental State Examination, *ADAS-cog* Alzheimer’s Disease Assessment Scale-cognitive subscale.

*Decline is the difference: value at M24 minus value at baseline; ^§^relative decline is the ratio (value at M24 minus value at M0)/value at M0.

### Plasmatic biomarkers level changes over time

As reported in Table 2, results show that TNF-R1 increases significantly over time, at 12 and 24 months, compared to baseline (p = 0.0018 and p < 0.0001, respectively). Conversely, TNFa decreases significantly at 24 months (M24) compared to baseline (p < 0.0001). Similar kinetics were observed for IL-6 (with a decrease at M24 compared to baseline, p = 0.0142), while its receptor (sIL-6R) decreases progressively over time (p < 0.0001 at 12 and 24 months versus baseline). IL-1b was higher at 12 months (M12, p = 0.0002) and 24 months (p = 0.0022) compared to baseline, while CCL5 and TIMP-1 remained stable at M24 compared to baseline. NFL was stable at M12 and increases significantly at M24 (p = 0.0052).

**Table 2:** Inflammatory markers levels at baseline, and at 12 and 24 months from the baseline.

Mean scores (SD) [min–max] pg/mL	Baseline	At 12-month-follow-up	At 24-month-follow-up	Global comparison P_Friedman_	12 months versus baseline	24 months versus baseline
TNF-R1 × 10^3^	2.8 (1.4) [1.2–10.7]	3.3(1.4) [1.7–9.4]	4.1 (2.0) [1.9–11.8]	< 0.0001	0.0018	< 0.0001
sIL-6R × 10^3^	36.3 (9.7) [22.7–68.2]	30.5 (8.0) [15.2–54.0]	27.1 (6.8) [15.4–45.6]	< 0.0001	< 0.0001	< 0.0001
TIMP-1 × 10^3^	339.2 (128.1) [166.6–753.1]	306.2 (107.4) [169.1–702.5]	333.8 (139.2) [163.9–755.4]	0.1388	0.0201	0.8402
IL-8	8.7 (6.2) [1.6–29.7]	9.7 (5.1) [3.5–26.4]	9.4 (5.9) [3.4–33.1]	0.0182	0.0645	0.3831
NFL	21.4 (11.4) [8.4–60.1]	20.9 (26.6) [7.7–67.0]	26.6 (19.0) [7.4–95.4]	0.0371	0.6374	0.0052
IL-1β	0.3 (0.3) [0.0–1.3]	0.9 (1.1) [0.0–5.7]	0.6 (0.6) [0.0–2.8]	< 0.0001	0.0002	0.0022
IL-6	1.4 (2.1) [0.0–35.1]	6.1 (18.8) [0.0–112.8]	0.7 (1.5) [0.0–6.9]	0.0304	0.5971	0.0142
TNFα	7.1 (6.4) [0.0–35.1]	17.3 (60.0) [0.0–363.1]	3.7 (2.9) [0.0–14.3]	< 0.0001	0.0756	< 0.0001
CCL5 × 10^3^	63.5 (88.8) [25.9–404.6]	67.1 (71.0) [161–332.1]	67.5 (116.5) [10.2–702.7]	0.2938	0.3823	0.8507

SD, Standard deviation; TNFR-1, tumor necrosis factor receptor 1; sIL-6R, soluble interleukin 6 receptor; TIMP-1, tissue inhibitor of metalloproteinases 1; IL-8, interleukin 8; NFL, neurofilament light protein; IL-1β, interleukin 1β; IL-6, interleukin 6; TNFα, tumor necrosis factor α; CCL5, chemokine ligand 5.

### Association between plasma biomarkers and cognitive decline

- Plasmatic cytokines: no correlation between plasmatic cytokine levels and variations in MMSE or ADASCog scores were found (Supplementary table 1). Moreover, baseline plasmatic cytokine levels did not differ between slow and fast decliners (Supplementary table 2).
- NFL: correlation was not significant between NFL value at baseline and cognitive decline during the follow-up (rho = 0.24; p = 0.1426 with ADAScog score and rho = − 0.20; p = 0.2166 with MMSE score) (Supplementary table 1). Correlations between NFL and ADAScog score were significant at M12 and M24 (rho = 0.32 p = 0.0490 and rho = 0.35, p = 0.0333, respectively). NFL and MMSE were also correlated at M24 (rho = − 0.45 p = 0.006). Same results were obtained after adjustment for all biomarkers studied (p < 0.05), except for IL-1 at M12 (p = 0.1426). No other correlation was found between NFL value and cognitive status. No significant association was found between changes in NFL during follow-up and changes in cognitive scores for both slow and fast decliners.

### Association between inflammatory markers and NFL

Significant correlations between NFL value and inflammatory markers are reported in Table 3. In particular, the correlation between NFL and TNF-R1 was observed at each time point during the follow-up (rho = 0.41, p = 0.0122 at M12 and rho = 0.69 p < 0.0001 at M24). A correlation between NFL and TNFa was also observed at baseline and at 24 months (rho = 0.33, p = 0.0433 and rho = 0.36 p = 0.0277, respectively). With respect to NFL kinetics and inflammatory parameters, a correlation was found between the variation of NFL and TNF-R1 between M12 and M24 (rho = 0.48; p = 0.005). Finally, NFL was correlated with IL-6, sIL-6R and IL-8 at baseline, and with TIMP-1 at baseline and at 12 months follow-up (Table 3).

**Table 3:** Correlations between NFL and inflammatory markers at baseline, 12 and 24 months of follow–up.

NFL correlation	Baseline		At 12 months-follow-up		At 24 months-follow-up	
	Rho	P	Rho	p	Rho	p
TNF-R1	0.60	0.0003	0.41	0.0122	0.69	< 0.0001
sIL-6R	0.48	0.0038	0.19	0.2441	0.26	0.1078
TIMP-1	0.57	0.0005	0.42	0.0107	0.18	0.2777
IL-8	0.39	0.0185	0.14	0.3789	0.40	0.0158
IL-1β	0.04	0.7944	0.09	0.5675	0.02	0.9017
IL-6	0.33	0.0433	0.10	0.5233	0.29	0.0824
TNFα	0.33	0.0468	0.13	0.4241	0.36	0.0277
CCL5	0.11	0.5119	0.04	0.8277	0.12	0.4562

TNF-R1, tumor necrosis factor receptor 1; sIL-6R, soluble interleukin 6 receptor; TIMP-1, tissue inhibitor of metalloproteinases 1 ; IL-8, interleukin 8 ; NFL, neurofilament light protein; IL-1β, interleukin 1β; IL-6, interleukin 6; TNFα, tumor necrosis factor α; CCL5, chemokine ligand 5.

## Discussion

In this study, we examined the level of expression of plasma biomarkers of inflammation (TNFa and its receptor [TNF-R1], IL-6 and its receptor [sIL-6R], CCL5, IL-8, IL-1b, TIMP-1) and NFL (highly informative of neuronal alteration), in 40 AD patients of Cytocogma cohort^32^, half of whom were considered to be slow decliner and half fast decliners (based on variation in MMSE over time). These patients were followed for 2 years, allowing us to study (1) the variation of plasma biomarker levels over time, (2) the relationship between plasma biomarker levels and the evolution of cognitive decline in AD patients.

The main result is represented by the strong correlation between TNF-R1 and NFL at the different follow-up time (baseline, 12 and 24 months). Our results also show a correlation between NFL and TNFa at baseline and at 24-month follow-up. This is the first time to our knowledge that such an association between plasma NFL and TNFa (or its receptor) is described, which may be promising for the prognosis of AD patients.

The activation of TNF-R1 is involved in amyloidogenic metabolism, in particular in the control of APP (Amyloid Precursor Peptide) processing and the level of Ab generation by the enzymatic activity and transcription of BACE1^34^. It is also thought to promote apoptosis by activating the NFκB pathway^35^. In addition, murine TNF-R1 has been shown to mediate Aβ neuronal death^36,37^. It should be noted that sTNF-R receptors are associated with conversion to dementia in subjects with mild cognitive impairment^38^.

Our present study highlights the progressive variation of some plasmatic biomarkers at 12 and 24 months from baseline: in particular TNF-R1 and NFL (increasing) and sIL-6R (decreasing). Plasmatic IL-6 and TNFa show particular kinetics, with an increase at 12 months from baseline, followed by a decrease at 24 months. No correlation between the inflammatory markers tested and cognitive status during this 2-year follow-up could be observed. A recent meta-analysis showed a significant difference between the level of inflammatory markers (such as IL-6, IL-1b, sTNF-R1 and 2) during cognitive decline, describing in particular an increase in their level in MCI and AD patients (compared to controls), both in CSF and blood^10,17^. We observe partly similar results in the blood, with increase of IL-6, IL-8 and sTNF-R1 at M12 and sTNF-R1 and IL-8 at M24, discrepancies regarding other biomarkers may rely on the cohort or on the sensitivity of methods used for their quantification. However, few studies have focused on the prognostic value of plasma cytokine levels on cognitive decline and the results remain controversial. In a previous study, we showed the absence of association between plasma levels of IL-1b, IL-6, TNFa or CCL5 and cognitive decline^33^: we confirm these data here and extend them with the same observation for sIL-6R, TIMP-1 or IL-8. Overall, these data suggest that, although the level of inflammation biomarkers in the blood may vary over time, their profile appears to have limited informative value on cognitive decline in patients with AD. However, the results need to be confirmed, particularly because of the limited number of patients in each group.

Interestingly, we found no association between NFL and cognitive decline in AD patients, which is in contradiction with previously published work^39,40^. This discrepancy may nevertheless be partly related to the limited size of our cohort. In addition, cognitive decline was assessed using MMSE and ADAScog, which are global cognitive assessments and therefore may not be sensitive enough to reflect the pathophysiological course of the disease. Thus, further investigation of a potential link between the value of NFL and other markers of disease progression (such as cortical atrophy or brain metabolism) in a larger cohort may provide more conclusive results.

Overall, the results of the current work likely suggest a role of peripheral inflammation in neuronal cell death and disease progression. Depending on our results and in particular the observed link between NFL, TNFa and TNF-R1, such mechanisms could involve the TNF/TNF-R pathway, which may open interesting perspectives for the prognosis of AD or therapeutic applications.

## Methods

### Patients

The current study is an ancillary study from the Cytocogma cohort. The details regarding the process of recruitment of this cohort have been previously described^32^. Briefly, patients were included in six French memory clinics between November 2010 and December 2012. Inclusion criteria were AD according to NINCDS-ADRDA criteria. All eligible patients underwent specific clinical evaluation and medical record review. Exclusion criteria were: neurological disease other than AD, ongoing inflammatory state (infection, neoplasia, or inflammatory disease), C-reactive protein (CRP) level greater than 10 mg/L, and any treatment with an anti-inflammatory drug. A total of 109 patients were included in the Cytocogma cohort.

All experimental protocols were approved by the ethics committee (‘comité de protection des personnes Ouest 3’). All methods were carried out in accordance with relevant guidelines and regulations. Informed consent was obtained from all subjects. The study is declared on ClinicalTrial.gov.NCT01351142.

All patients met the NINCDS-ADRDA criteria, with a MMSE score between 16 and 25. Patients with confounding inflammatory factors were excluded. Patients were followed for 2 years; 20 patients with the fastest cognitive decline and 20 with the lowest cognitive decline were selected within this cohort. Categorization into fast or slow decliners was carried out according to MMSE relative variation during the follow up. Patients with a relative change in MMSE inferior to the 25th percentile were considered as slow decliners. Patients with a relative change in MMSE superior to the 25th percentile were considered as fast decliners.

Length of disease progression of the patients was evaluated by the interval between first symptoms and inclusion.

### Biological assessment

Measures of IL-1β, IL-6, TNFα and CCL5 (RANTES) were performed as previously described^33^, using Luminex custom kit. Concentration was calculated using the best parameter logistic fit curve generated from the seven standards using Milliplex™ Analyst Software.

Simplex methods were performed for the quantification of plasmatic biomarkers of interest, using either electrochemiluminescence (MesoScaleDiscovery technology, MSD, Sector Imager 2400A) for TNF-R1 (ref K151BIC-1), ELISA method (kits purchased from Clinisciences) for sIL-6R and TIMP-1 or Quanterix methods for IL-8 and NFL (ref: 100198 and ref: 102258 , respectively). Samples were quantified in duplicate and depending on the kits used, samples were either used pure or diluted 1:20, 1:50 or 1:500.

Plasmatic biomarker assessment were realized at inclusion and after 1 and 2 years of follow-up.

### Statistics

All statistical analyses were carried out using the SAS 9.2 software package (SAS Inc., Cary, NC, USA). Quantitative variables are described with mean and standard deviation (SD) and extreme values [minimum–maximum]. Qualitative variables were expressed as number and percentage.

Cognitive decline was calculated as the difference of MMS or ADAScog scores between M24 value and baseline value (or between M12 value and baseline value if specified).

Fast and slow decliners were compared using Chi2 test for qualitative variables or Student *t* test or non parametric unpaired Wilcoxon test for quantitative variables.

Changes of plasmatic biomarkers levels over time were tested between the 3 time-points baseline, 12 months and 24 months using non parametric Friedman test. They were also tested between baseline and 12 months and between baseline and 24 months using non parametric paired Wilcoxon test with a Bonferroni correction setting the alpha value for each comparison equal to 0.025.

Correlations between biomarkers and scores and between inflammatory markers and NFL were assessed by Spearman coefficient correlation (rho).

Multiple comparisons were accounted for using a lower p value (p < 0.01) in most cases. In some analysis, however, the p value was considered equal to 0.05, given the exploratory nature of the analysis, and no correction for multiple comparisons was then made.

## Supplementary Information

Supplementary Tables.
