# Supplementary material for: NFL strongly correlates with TNF-R1 in the plasma of AD patients, but not with cognitive decline
Source: Sci Rep. 2021 May 13;11:10283. doi: 10.1038/s41598-021-89749-5 (PMC8119968; doi:10.1038/s41598-021-89749-5)
Supplement: Supplementary file 1 — Supplementary Tables. [file 41598_2021_89749_MOESM1_ESM.docx]

**Supplementary Tables:**

**Supplementary table 1 : Correlations between inflammatory markers at baseline and cognitive decline (MMSE or ADASCog scores)**

| M0 | MMS score at M0 | | ADADScog score at M0 | | 12 months MMS variation from baseline | | 24 months MMS variation from baseline | | 12 months ADAScog variation | | 24 months ADAScog variation | |
| --- | --- | --- | --- | --- | --- | --- | --- | --- | --- | --- | --- | --- |
|  | Rho | P | Rho | P | Rho | p | Rho | P | Rho | p | Rho | p |
| TNF-R1 | -0.30 | 0.0642 | 0.16 | 0.3115 | 0.10 | 0.5394 | 0.05 | 0.7431 | -0.17 | 0.2775 | -0.03 | 0.8699 |
| sIL-6R | -0.32 | 0.0479 | 0.06 | 0.6850 | -0.10 | 0.5279 | -0.07 | 0.6504 | 0.08 | 0.6084 | 0.12 | 0.4440 |
| TIMP-1 | -0.16 | 0.3204 | 0.13 | 0.4185 | -0.10 | 0.5269 | -0.15 | 0.3585 | 0.11 | 0.4990 | 0.18 | 0.2700 |
| IL-8 | 0.15 | 0.3501 | -0.08 | 0.6337 | -0.19 | 0.2456 | -0.15 | 0.3513 | 0.21 | 0.1925 | 0.08 | 0.6181 |
| IL-1β | 0.091 | 0.5681 | -0.26 | 0.0988 | 0.15 | 0.3427 | 0.02 | 0.7944 | -0.02 | 0.9049 | -0.12 | 0.4355 |
| IL6 | -0.15 | 0.3496 | 0.27 | 0.0895 | 0.01 | 0.9521 | 0.33 | 0.8849 | -0.08 | 0.5962 | -0.06 | 0.7031 |
| TNFα | 0.003 | 0.5681 | 0.10 | 0.5441 | 0.03 | 0.8433 | -0.04 | 0.8159 | -0.07 | 0.6408 | -0.07 | 0.6416 |
| CCL5 | -0.01 | 0.9661 | -0.08 | 0.6023 | -0.09 | 0.5909 | 0.16 | 0.3152 | -0.002 | 0.9890 | -0.16 | 0.3307 |
| NFL | -0.20 | 0.2166 | 0.24 | 0.1426 | -0.26 | 0.1128 | -0.24 | 0.1513 | 0.06 | 0.7180 | 0.07 | 0.6891 |

Variation of cognitive score was calculated as score at 24 months minus score at baseline

**Supplementary table 2 : Comparison of baseline inflammatory markers levels in fast decliners and slow decliners**

| Mean (SD) or median [min-max] pg/mL | Fast decliners (n=20) | Slow decliners (n=20) | p |
| --- | --- | --- | --- |
| TNF-R1 .10^3^ | 2.8 (1.6) | 2.8 (1.3) | 0.787 |
| sIL-6R .10^3^ | 38.1 (11.5) | 34.6 (7.4) | 0.372 |
| TIMP-1 .10^3^ | 350.9 (144.6) | 327.5 (111.9) | 0.675 |
| IL-8 | 9.0 (6.6) | 8.5 (6.0) | 0.636 |
| NFL | 22.2 (11.5) | 20.7 (11.7) | 0.465 |
| IL-1β | 0.2 [0.0-1.3] | 0.2 [0.0-1.0] | 0.957 |
| IL-6 | 0.7 [0.0-8.8] | 0.7 [0.0-7.1] | 0.935 |
| TNFα | 5.8 [0.0-35.1] | 5.1 [0.0-22.2] | 0.829 |
| CCL5 .10^3^ | 23.9 [0.03-231.8] | 24.5 [1.6-404.7] | 0.204 |
